# Dysregulation of RasGRP1 in rheumatoid arthritis and modulation of RasGRP3 as a biomarker of TNFα inhibitors

**DOI:** 10.1186/s13075-015-0894-9

**Published:** 2015-12-26

**Authors:** Marie-Laure Golinski, Thibault Vandhuick, Céline Derambure, Manuel Fréret, Matthieu Lecuyer, Clément Guillou, Martine Hiron, Olivier Boyer, Xavier Le Loët, Olivier Vittecoq, Thierry Lequerré

**Affiliations:** INSERM, U905 & Normandy University, Institute for Research and Innovation in Biomedicine (IRIB), Rouen, France; Department of Rheumatology & CIC/CRB 1404, Rouen University Hospital, Rouen, France; NeoVasc ERI 28 & Normandy University, Institute for Research and Innovation in Biomedicine (IRIB), Rouen, France; Department of Immunology, Rouen University Hospital, Rouen, France; INSERM U905, Université de Rouen, Faculté de médecine - pharmacie, 22 boulevard Gambetta, 76000 Rouen, France

**Keywords:** Rheumatoid arthritis, T cells, B cells, RasGRP, TNFα inhibitors

## Abstract

**Background:**

B and T cells play a key role in rheumatoid arthritis (RA) pathophysiology. RasGRP1 and RasGRP3 are involved in T and B cell receptors signaling, and belong to gene combination able to predict infliximab responsiveness, leading to the question of RasGRP1 and RasGRP3 involvement in RA.

**Methods:**

RasGRP1 and RasGRP3 expression levels were measured by qRT-PCR and/or western-blot in peripheral blood mononuclear cells (PBMCs), in T and B cells from untreated RA patients and in RA patients treated by TNFα inhibitors. T and B cells from healthy controls (HC) were cultured with TNFα, and TNFα receptors neutralizing antibodies to highlight the TNFα effects on RasGRP1 and RasGRP3 pathways. MAPK pathways and apoptosis were respectively analyzed using the Proteome Profiler arrays and flow cytometry.

**Results:**

In PBMCs from RA patients, gene expression levels of *RasGRP1* were invariant while *RasGRP3* was downregulated under TNFα inhibitors and upregulated under TNFα. In T cells from RA patients, RasGRP1 was decreased and its gene expression level was correlated with disease activity. In T cells from HC, TNFα stimulation increased *RasGRP1* gene expression level while it reduced RasGRP1 protein expression level. Bryostatin-1 experiments have confirmed that the TNFα effect observed on T cells proliferation was due to the decrease of RasGRP1 expression. Besides, *RasGRP3* expression level increased in PBMCs from RA patients under TNFα and in B cells from HC leading us to conclude that RasGRP3 in B cells was modulated by TNFα.

**Conclusion:**

This study demonstrates RasGRP1 dysregulation in RA patients while RasGRP3 is characterized as a biomarker linked to TNFα inhibitors. After binding to TNFR1, TNFα reduced RasGRP1 protein expression resulting in inhibition of T cell activation.

**Trial registration:**

Clinicaltrials.gov NCT00234234, registered 04 November 2008; NCT00767325, registered 05 October 2005.

**Electronic supplementary material:**

The online version of this article (doi:10.1186/s13075-015-0894-9) contains supplementary material, which is available to authorized users.

## Background

Rheumatoid arthritis (RA) is the most common inflammatory arthritis affecting 1 % of the world population [1]. RA is characterized by chronic inflammation and proliferation of the synovial tissue, leading to the destruction of bone and cartilage. RA is a multifactorial disease involving genetic, environmental and hormonal factors, and immunological disorders contributing to chronic synovitis [2].

B and T lymphocytes play a central role in RA [3]. Some abnormalities of antigen receptor signaling pathways in B and T cells can lead to their dysfunction, particularly by enhancing the emergence of autoimmunity [4]. The dysfunction of various immune cells is a result of an imbalance in the production of factors which leads to the overexpression or conversely, the deletion of other factors, such as antibodies and cytokines. Among these factors, TNFα has been identified as a key cytokine in the pathogenesis of RA maintaining joint inflammation [5, 6]. Indeed, the use of anti-TNFα drugs has allowed eliciting remission in a high proportion of RA patients even if 30 % of patients do not respond to them [7]. Studies have been performed to highlight predictive biomarkers of response to TNFα inhibitors, including *RasGRP3* [8]. *RasGRP3* has also been found to be dysregulated in peripheral blood mononuclear cells (PBMCs) and synovium from RA patients [8, 9]. Furthermore, *RasGRP1* has been associated with susceptibility to RA [10].

RasGRP is a member of the CDC25 family of ras guanyl nucleotide exchange factors that contain an N-terminal GEF domain and C-terminal calcium-binding and diacylglycerol (DAG)-binding domains [11]. In mouse, RasGRP3 is expressed in B cells whereas RasGRP1 is highly expressed in T cells and to a lesser extent in B cells [12–16]. These proteins are involved in T and B cell receptor (respectively TCR and BCR) signaling [17, 18]. RasGRP1 also plays a role in NF-κB pathway inhibition in B cells, leading to their apoptosis [19]. Ras activation by RasGRP proteins stimulates various effectors systems, leading to changes in gene expression that are critical for T or B cell development [20–22]. Indeed, *RasGRP1*^*-/-*^ mice become autoimmune-prone and develop a lupus-like phenotype [20, 22, 23]. These mice displayed an increase of autoreactive CD4^+^ T cells, which is the consequence of a lack of positive selection in the thymus, thus facilitating the activation of B cells and the production of auto-antibodies (Ab) [12, 13]. In contrast, *RasGRP3*^*-/-*^ mice exhibit hypogammaglobulinemia and show no sign of autoimmunity [12, 20]. Remarkably, double mutant mice do not develop signs of autoimmunity [12]. Therefore, RasGRP1 inhibition promotes autoimmunity via activation of B cells by autoreactive CD4^+^ T cells, while RasGRP3 inhibition renders B cells less sensitive to T cell signals [20].

The identification of *RasGRP3* as a biomarker of anti-TNFα drugs raises the question as to whether RasGRP is a biomarker related to RA pathology or to the treatment. We therefore investigated *RasGRP1* and *RasGRP3* gene expression in patients treated by two TNFα inhibitors, adalimumab and etanercept, and in untreated RA patients compared to healthy controls (HC).

## Methods

### Subjects

A total of 60 patients (adalimumab (n = 21), etanercept (n = 9) or abatacept (n = 30)) were included to measure the impact of biologic agents on RasGRP1 and RasGRP3 expression levels (Additional file 1: Table S1). Patients treated with adalimumab or etanercept fulfilling the 1987 American College of Rheumatology (ACR) or the 2010 ACR/European League Against Rheumatism (EULAR) criteria for RA were included in the multicenter SATRAPE study (NCT00234234), approved by the ethics committee of Upper-Normandy in France (n°2005/006) [24, 25]. RA patients treated with abatacept, who were used as controls came from the APPRAISE study (NCT00767325) approved by the ethics committee of CPP (Comité de Protection des Personnes) in France [26]. RA patients were treated as recommended by the manufacturer and the French Drug Agency ANSM (50 mg every week for etanercept, 40 mg each other week for adalimumab patients by subcutaneous injections and 10 mg/kg every month by intravenous injections for abatacept). Clinical and biological characteristics such as age, gender, tender and/or swollen joint count, disease activity score (DAS28), treatments and their dose, health assessment questionnaire, serum C-reactive protein level and erythrocyte sedimentation rate, were recorded just before the first injection and 3 months later.

To compare RasGRP1 and RasGRP3 expression levels in RA patients and HC, 20 HC (6 male and 14 female; 32 ± 9 years old) and 32 untreated RA patients (9 male and 23 female; 53 ± 15 years old) were studied (Additional file 2: Table S2). At the time when RasGRP1 and RasGRP3 expression levels were measured, DAS28 was 4.98 ± 1.32. The PBMCs from RA patients or HC were collected from whole venous blood. All participants signed an informed consent at the time of enrollment.

PBMCs were isolated from the buffy-coat of HC to perform in vitro studies.

### Purification of T and B cells

PBMCs were extracted from whole venous blood or buffy coat using Ficoll-Hypaque (Lymphoprep™, Oslo, Norway). T and B cells were purified by negative selection using human T and B cell Dynabeads, respectively, according to the manufacturer’s instructions (Invitrogen™, Carlsbad, CA, USA).

### Lymphocyte cultures

One million PBMCs, T or B cells/well were cultured into 24-well plates with 1 ml of RPMI 1640 medium supplemented with 10 % fetal calf serum (FCS), and 0.5 % penicillin and streptomycin. PBMCs or lymphocytes were cultured with or without 1 ng/ml of TNFα (R&D Systems, Minneapolis, MN, USA) and with or without adalimumab (1 μg/ml), etanercept (10 μg/ml), infliximab (100 μg/ml), certolizumab (1 μg/ml) or golimumab (100 μg/ml) for 24 or 48 hours in a 5 % CO_2_ incubator at 37 °C. In neutralizing experiments, B and T cells were preincubated with anti-TNF receptor (TNFR)1 (9 μg/ml) or anti-TNFR2 (2.5 μg/ml) (R&D Systems) for 1 hour and TNFα was added for 48 hours. In activation experiments, T cells were incubated with IL-2 (60 U/ml) and anti-CD3 antibody (5 μg/ml) for 4 days. For experiments with Bryostatin-1, cells were incubated with TNFα for 48 hours. Cells were then activated with 50 nM Bryostatin-1 (Sigma-Aldrich, Saint Louis, MO, USA) for 5 hours. Cells were then harvested for flow cytometry analysis or RNA and protein extraction.

### Flow cytometry analysis

To control the purity of cell selection, the following antibodies (BD Pharmingen™, Franklin Lakes, NJ, USA) were used: anti-CD3, anti-CD4, anti-CD8, anti-CD14, anti-CD19 and anti-CD56. Antibodies used for TNF receptor expression (anti-TNFR1 and anti-TNFR2) were purchased from R&D Systems (R&D Systems). To bring out the T cell activation, the following antibodies (Beckman Coulter, Brea, CA, USA) were used: anti-CD3, anti-CD25, anti-HLA-DR. To bring out the early T cell activation, the marker CD69 was studied after TNFα stimulation by flow cytometry (BD Pharmingen™). To detect apoptosis in T cells after TNFα stimulation, a fluorescein isothiocyanate (FITC) annexin-V Apoptosis Detection Kit (Becton-Dickinson, Franklin Lakes, NJ, USA) was used. Appropriate isotype controls were used in all cases. Acquisition of samples was performed on a FACSCanto flow cytometer (Becton-Dickinson), and the data were analyzed with FlowJo software.

### Quantitative measurement of *RasGRP1* and *RasGRP3* mRNA abundance

RNA samples were obtained using the RNAqueous 4-PCR kit according to the manufacturer’s instructions (Ambion®, Austin, TX, USA). cDNA was then synthesized using random primers and M-MLV enzyme (Invitrogen™). Quantitative (q)RT-PCR was performed using a Lightcycler as instructed by the manufacturer (Roche™, Meylan, France). We chose primers that recognize normal and abnormal *RasGRP1* mRNA. Sequences of primers used for qRT-PCR were : *RasGRP1* forward, 5′-TGCACCGAATTGTCATCTCC-3′; *RasGRP1* reverse, 5′-GTCAATCAGGCGGCAATGTA-3′; *RasGRP3* forward, 5′-CACGGTCATCAACAAGCACA-3′; *RasGRP3* reverse, 5′-CAGTGTTCGCAGAAGGTTGG-3′; *18S* forward, 5′-GTGGAGCGATTTGTCTGGTT-3′; *18S* reverse, 5′-CGCTGAGCCAGTCAGTGTAG-3′ (Eurogentec™, Fremont, CA, USA). qRT-PCR reactions for each sample were performed in triplicate using SYBR-Green and values corrected using control 18S to calibrate (Roche™).

### Lymphocyte proliferation assay

Cell proliferation is evaluated by incorporation of (^3^H)-thymidine. Fifty thousand cells/well of B or T cells in 200 μl of RPMI containing 10 % FCS were seeded into 96-well plates. Lymphocytes were cultured with or without TNFα (for 48 and 72 hours) in a 5 % CO_2_ incubator at 37 °C. After these incubations, 1 μCi of (^3^H)-thymidine was added to each well. (^3^H)-thymidine incorporation was quantified 16 hours later.

### Signaling pathway analysis

A human phospho-kinase Proteome Profiler™ array (R&D Systems) was used to investigate the mitogen-activated protein kinase (MAPK) signaling pathways. To conduct this experiment, B and T cells were rinsed twice with PBS, and lysis buffer was added. Cell lysates were gently rocked for 30 minutes at 4 °C and then centrifuged at 14,000 g for 5 minutes (4 °C). A total of 200 μg of protein was used for each array. Human phospho-kinase was purchased from R&D Systems. Proteins were visualized using a chemiluminescence detection system. Arrays were exposed to x-ray films (5–45 minutes) and developed under standard conditions. The intensity of the immunoreactive bands was quantified using a blot analysis system (BioRad Laboratories, Hercules, CA, USA).

### Immunoblot analysis

B and T cells were incubated with RIPA supplemented with a protease inhibitor for 30 minutes. Homogenates were then centrifuged at 16,000 g for 10 minutes at 4 °C. The protein concentration of supernatant was quantified using the bicinchoninic acid kit (Pierce Biotechnology, Rockford, IL, USA). Lysates were suspended in Laemmli buffer (100 mM Hepes, pH 6.8, 10 % β-mercaptoethanol, 20 % SDS) and boiled for 10 minutes. Proteins (15 to 40 μg) were loaded and resolved on 12 % SDS-PAGE gels. After electrophoretic separation at 200 V for 1 hour, proteins were transferred to a polyvinylidene difluoride membrane at 30 V for 2 hours. Membranes were then incubated in a blocking solution (5 % BSA in Tris-buffered saline containing 0.1 % Tween 20) at room temperature for 1 hour and incubated overnight with one of the following primary antibodies: mouse anti-human-RasGRP1 (Merck Millipore, Molsheim, France), mouse anti-human-RasGRP3 (Abcam, Cambridge, MA, USA), mouse anti-human-extracellular signal-regulated kinase (ERK)1/2 (Merck Millipore), mouse anti-human-phospho-ERK1/2 (Cell Signaling Technology®, Danvers, MA, USA) or mouse anti-human-glyceraldehyde-3-phosphate dehydrogenase (GAPDH) (Sigma-Aldrich). After incubation with the corresponding secondary antibodies coupled to peroxidase (Santa-Cruz Biotechnology, Santa-Cruz, CA, USA), proteins were visualized using enhanced chemiluminescence ECL immunoblotting detection system (Amersham, UK). Arrays were exposed to x-ray films (5–20 minutes) and developed under standard conditions. The intensity of the immunoreactive bands was quantified using a blot analysis system (BioRad Laboratories). Results are reported relative to GAPDH.

### Statistical analysis

One-way analysis of variance (ANOVA) followed by Dunnett post-hoc test was used to compare *RasGRP1* and *RasGRP3* gene expression levels between different groups of patients and HC and for the proliferation assay. One-way ANOVA followed by Bonferroni post-hoc test was used to compare *RasGRP1* and *RasGRP3* gene expression levels with or without TNFα and TNFα inhibitors in HC, and RasGRP1 and RasGRP3 expression levels with or without TNFα and anti-TNFR treatment. The nonparametric Wilcoxon paired test was used to compare baseline and post-treatment *RasGRP1* and *RasGRP3* gene expression levels. Comparison of two populations was made with Student’s *t* test. Correlations were assessed using Pearson’s rank correlation coefficient.

## Results

### *RasGRP3* gene expression level decreased in PBMCs from RA patients treated by TNFα inhibitors

**Fig. 1:**
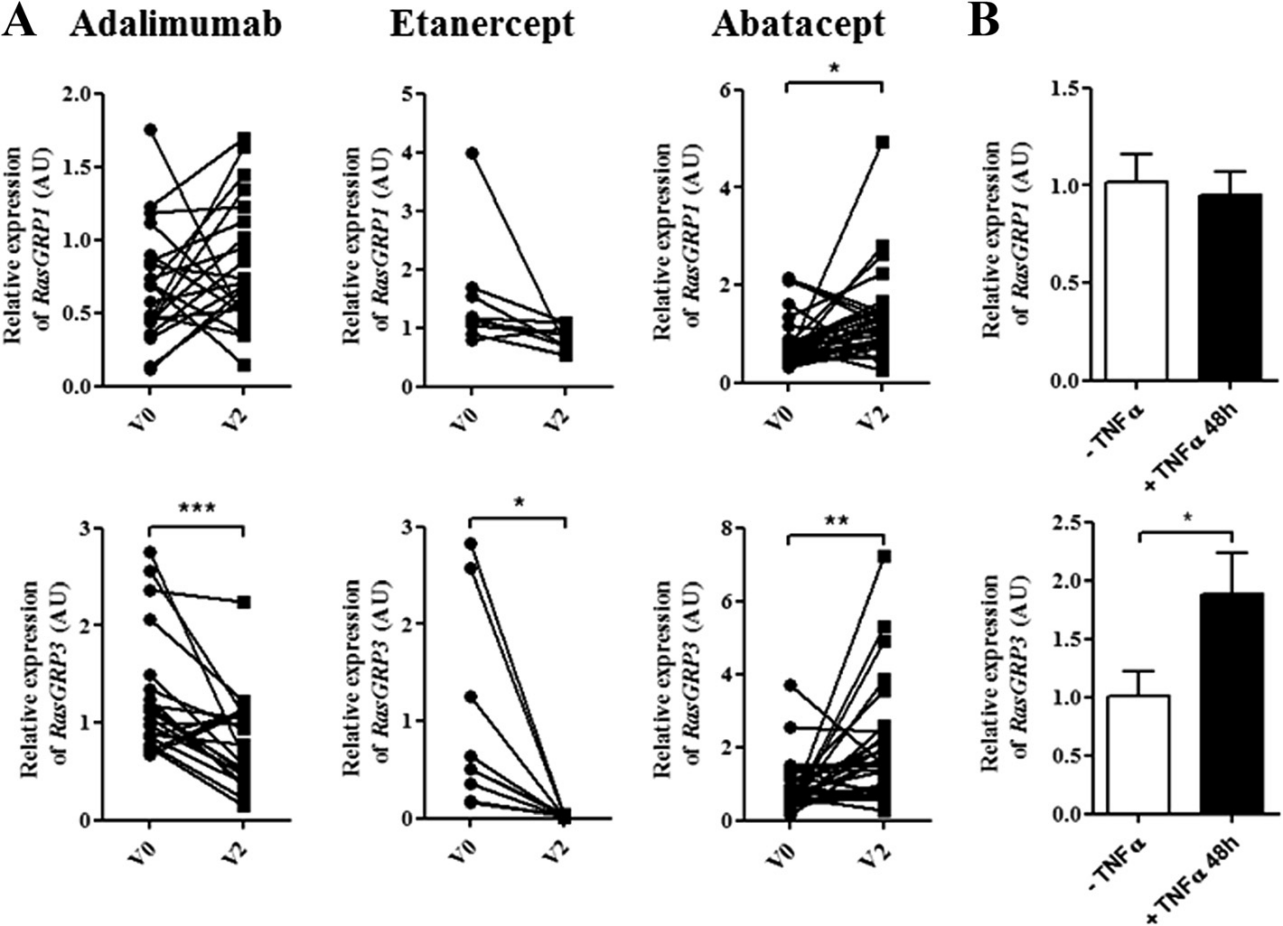
*RasGRP1* and *RasGRP3* gene expression fluctuations in periperhal blood mononuclear cells (PBMCs) from patients with rheumatoid arthritis (RA) treated with biologic agents at baseline and after treatment or after in vitro TNFα stimulation. **a** PBMCs were isolated from whole blood from RA patients treated with adalimumab (n = 21; 4 male and 17 female; 52 ± 3 years old) and etanercept (n = 9; 4 male and 5 female; 54 ± 5 years old) before treatment (V0) and 3 months later (V2) or abatacept (n = 30; 3 male and 27 female; 61 ± 1 years old) before treatment (V0) and 6 months later (V2). **b** PBMCs were isolated from whole blood of RA patients untreated with biologics (n = 6; 1 male and 5 female; 54 ± 10 years old) and cultured with or without TNFα for 24, 48 or 72 hours. Quantitative PCR analysis was performed to measure *RasGRP1* and *RasGRP3* gene expression levels. The relative expression levels (in arbitrary units (AU)) of *RasGRP1* and *RasGRP3* were normalized with *18S* RNA abundance. Mean ± standard error of the mean were compared using Student’s *t* test or the Wilcoxon paired test: *p <0.05; **p <0.01; ***p <0.001

To better understand the effects of TNFα inhibitors on *RasGRP3*, gene expression levels in PBMCs from RA patients were compared before and after 3 months (V2) of treatment with an antibody targeting TNFα (adalimumab) or a soluble receptor of TNFα (etanercept), or after 6 months of treatment with abatacept (CTLA4-Ig fusion protein), another family of biologic agent used in RA as a control. *RasGRP3* gene expression level was significantly decreased under adalimumab (*p* <0.001) or etanercept (*p* <0.05) after 3 months of treatment (Fig. 1a). RasGRP is also involved in immune processes, but the *RasGRP1* gene expression level was not significantly dysregulated under TNFα inhibitors (Fig. 1a). Otherwise, *RasGRP1* (*p* <0.05) and *RasGRP3* (*p* <0.01) gene expression levels were significantly increased in patients after 6 months of treatment with abatacept (Fig. 1a).

To highlight the effects of all TNFα inhibitors available on *RasGRP3* gene expression levels in in vitro conditions, PBMCs from HC were incubated with or without TNFα and TNFα inhibitors for 1.5, 6.0, 24.0 and 48.0 hours. TNFα induced a significant increase of *RasGRP3* gene expression levels only after 48.0 hours of stimulation (Additional file 3) while any modulation was observed at early time: 1.5, 6.0 and 24.0 hours (data not shown). This increase of *RasGRP3* gene expression level was inhibited by all TNFα inhibitors (Additional file 3). As expected, no effect on *RasGRP3* gene expression level in PBMCs was observed when TNFα inhibitors were added to the cultures without TNFα (data not shown). All together, these results indicate that the decrease of *RasGRP3* gene expression level is specifically associated with all TNFα inhibitors, raising the question about the effects of TNFα on RasGRP3 expression in RA patients.

### *RasGRP3* gene expression level increased in PBMCs from RA patients stimulated by TNFα

To investigate the involvement of TNFα in *RasGRP1* and *RasGRP3* gene expression levels in PBMCs from RA patients, these cells were incubated with or without TNFα for 48 hours. TNFα induced a significant increase of *RasGRP3* gene expression level (*p* <0.05) (Fig. 1b) as observed in PBMCs from HC (Additional file 3), without an effect on *RasGRP1* gene expression level (Fig. 1b).

### *RasGRP1* and *RasGRP3* were respectively expressed in T and B cells from HC

**Fig. 2:**
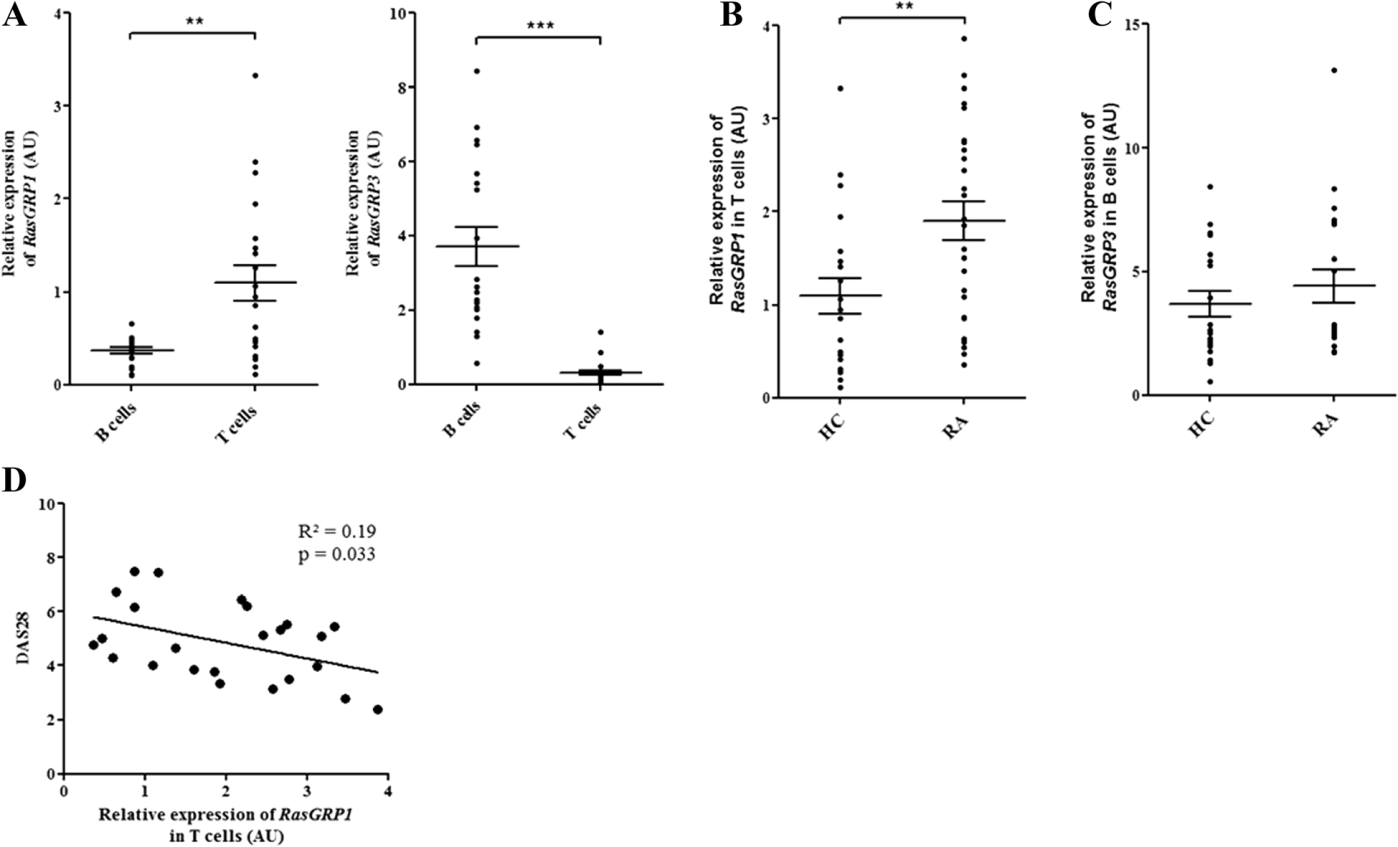
*RasGRP1* and *RasGRP3* gene expression levels in B and T cells from healthy controls and patients with rheumatoid arthritis (RA). After T and B cell negative selection, quantitative PCR analysis of *RasGRP1* and *RasGRP3* gene expression was performed. **a** *RasGRP1* and *RasGRP3* gene expression levels in B and T cells from healthy controls (HC) (n = 20; 6 male and 14 female; 32 ± 9 years old). **b** *RasGRP1* and **c** *RasGRP3* gene expression levels respectively in T and B cells from HC (n = 20; 6 male and 14 female; 32 ± 9 years old) and patients with RA (n = 26; 8 male and 18 female; 53 ± 15 years old). **d** Correlation between *RasGRP1* gene expression level in T cells and RA disease activity (disease activity score in 28 joints (DAS28)) was assessed using Pearson’s rank correlation coefficient. The relative expression levels (in arbitrary units (AU)) of *RasGRP1* and *RasGRP3* were normalized with *18S* RNA abundance. Mean ± standard error of the mean were compared using the Wilcoxon paired test or one-way analysis of variance followed by Dunnett post-hoc test. * p < 0.05; ** p < 0.01; *** p < 0.001

While a few studies have described RasGRP1 and RasGRP3 expression on T and B cells in mice or in cell lines [12, 21], several studies have only described the expression of RasGRP1 in T cells [27–30], raising the question of RasGRP3 expression in human. After negative selection, the B and T cell purity was 91 % (±0.1 %) and 96 % (±0.02 %), respectively (Additional file 4). In HC, *RasGRP1* gene expression level was significantly higher in T cells than in B cells (*p* <0.01). Conversely, *RasGRP3* gene expression level was significantly higher in B cells than in T cells (*p* <0.001) (Fig. 2a). *RasGRP1* and *RasGRP3* were unexpressed respectively in B and T cells.

### RasGRP1 was dysregulated in T cells from RA patients

*RasGRP1* and *RasGRP3* gene expression levels were next measured in T and B cells from RA patients compared to HC. *RasGRP1* gene expression level was significantly overexpressed in T cells from RA (*p* <0.01) (Fig. 2b) while *RasGRP3* gene expression level in B cells was not significantly different between RA patients and HC (Fig. 2c). To link RasGRP1 or RasGRP3 with RA, we assessed the correlation between DAS28 and gene expressions. *RasGRP1* gene expression level in T cells was inversely correlated with the RA disease activity measured by DAS28 in 24 RA patients (*p* <0.05) (Fig. 2d). No correlation between *RasGRP3* gene expression level and DAS28 in B cells was found (data not shown).

To determine whether the increasing of *RasGRP1* mRNA expression level was due to a modification of RasGRP1 function/signaling or if it reflected the activation status of the T cells, T cells were activated for 4 days with IL-2 and anti-CD3 antibody. First, we confirmed that T cells activation markers HLA-DR and CD25 were overexpressed after 4 days of stimulation by flow cytometry (Additional file 5A). Second, *RasGRP1* gene expression level was significantly increased (*p* <0.001) after T cell activation (Additional file 5B).

**Fig. 3:**
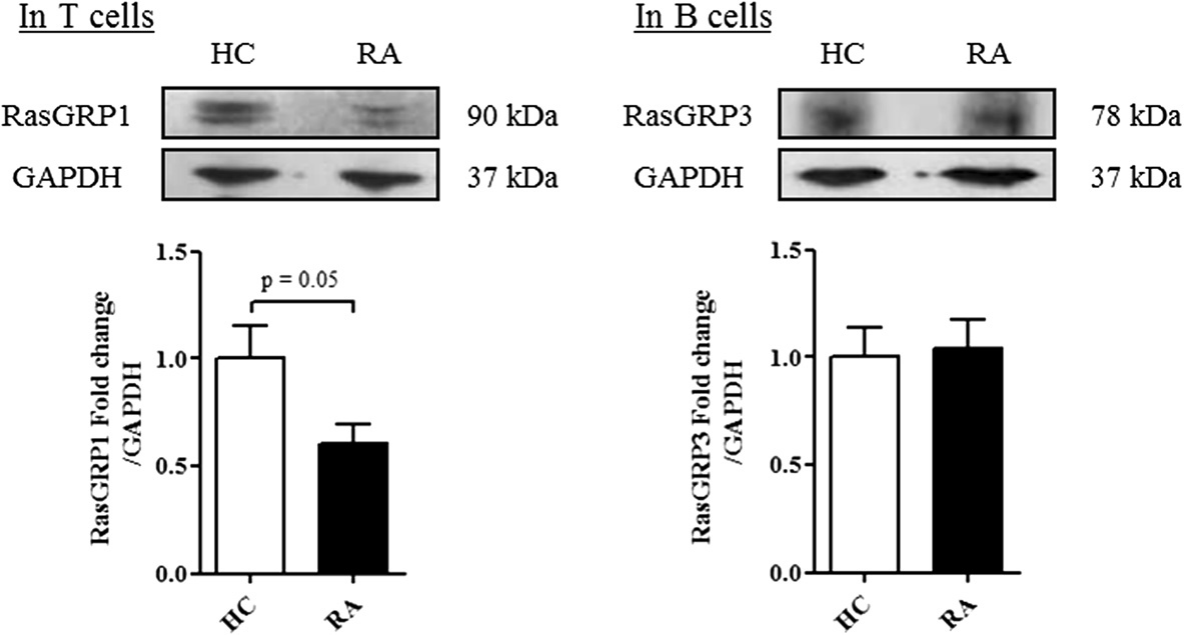
RasGRP1 and RasGRP3 protein expression levels in T and B cells in healthy controls (HC) and rheumatoid arthritis (RA) patients. Western blot analysis was performed to measure RasGRP1 and RasGRP3 protein expression in T and B cells, respectively. RasGRP1 protein expression was measured in T cells from HC (n = 4) and RA patients (n = 5). RasGRP3 protein expression was measured in B cells from HC (n = 4) and RA patients (n = 3). A value of 1 was arbitrarily assigned to control conditions to which glyceraldehyde-3-phosphate dehydrogenase (GAPDH) was reported and expressed as fold change. Mean ± standard error of the mean were compared using Student’s *t* test

To confirm these results at the protein level, RasGRP1 and RasGRP3 expression level were measured by western blot respectively in T and B cells from RA patients compared to HC. Unexpectedly, the expression of *RasGRP1* in T cells from RA patients was 50 % lower (*p* = 0.05) while RasGRP3 protein expression level in B cells was not significantly different (Fig. 3). Altogether these results indicate that RasGRP1 is associated with RA disease activity, whereas RasGRP3 was not involved in RA pathophysiology. Surprisingly, the gene and protein RasGRP1 expression changed in the opposite sense in RA patients.

### TNFα influences *RasGRP1* and *RasGRP3* gene expression levels respectively in T and B cells

**Fig. 4:**
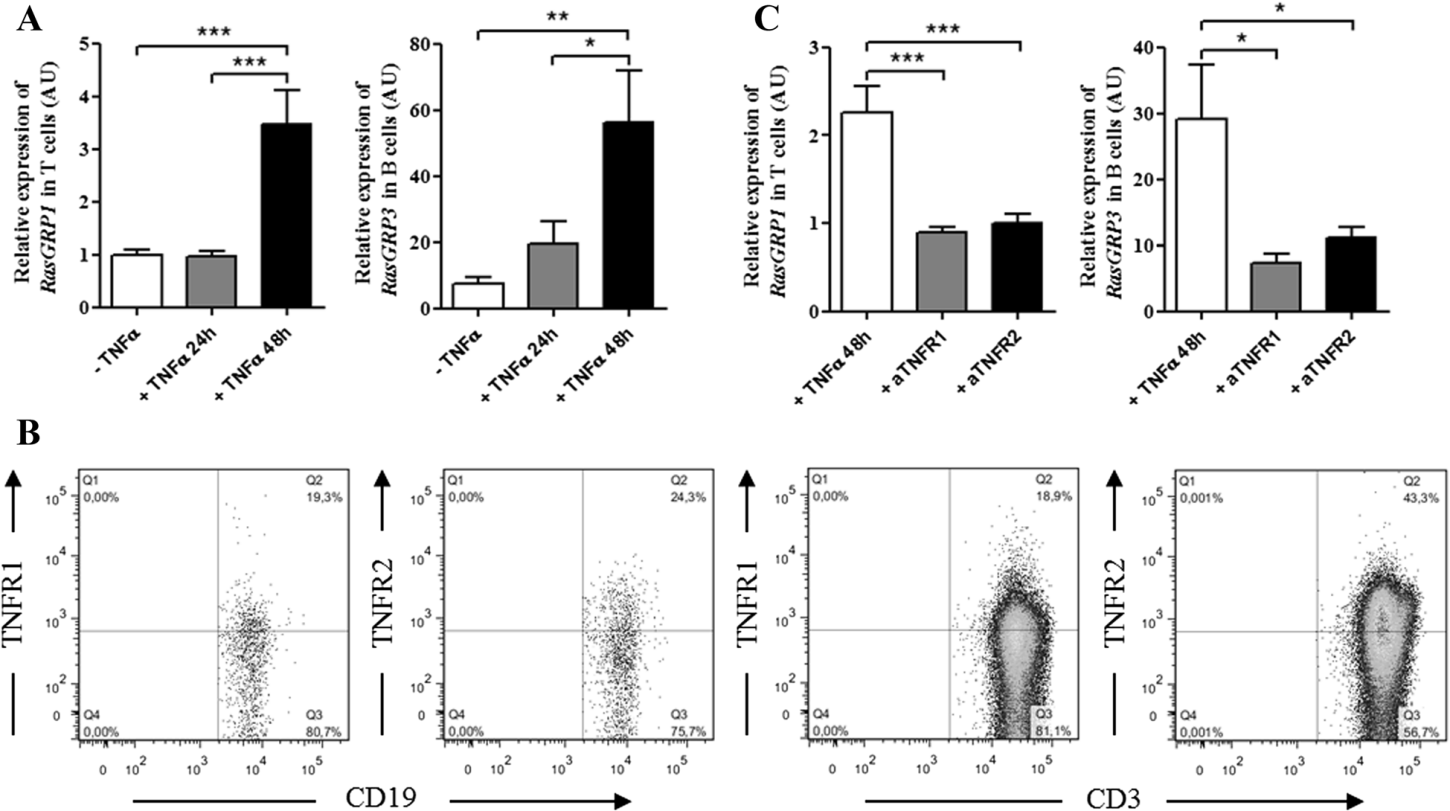
TNFα influences *RasGRP1* and *RasGRP3* gene expression levels in T and B cells respectively *via* TNFR1 and TNFR2. **a** Quantitative PCR analysis was performed to measure *RasGRP1* and *RasGRP3* gene expression levels in T and B cells, respectively, obtained from three buffy coats. In each condition, cells were cultured with or without TNFα for 24 and 48 hours. **b** TNF receptor (TNFR)1 and TNFR2 expression in B (CD19) and T cells (CD3) was checked by flow cytometry. **c** Quantitative PCR analysis was performed to measure *RasGRP1* and *RasGRP3* gene expression levels in T and B cells, respectively, from three buffy coats. In each condition, T or B cells, previously exposed to anti-TNFR1 or anti-TNFR2 neutralizing antibodies, were cultured with TNFα for 48 hours. The relative expression levels (in arbitrary units (AU)) of *RasGRP1* and *RasGRP3* were normalized with *18S* RNA abundance. Mean ± standard error of the mean were compared using one-way analysis of variance followed by Bonferroni post-hoc test: *p <0.05; **p <0.01; ***p <0.001

In RA, TNFα is a key pro-inflammatory cytokine involved in RA pathophysiology and is also a therapeutic target. To better understand the involvement of TNFα on *RasGRP1* and *RasGRP3* gene expression levels on T and B cells, respectively, these cells from buffy coats were incubated with or without TNFα for 24 or 48 hours. TNFα induced an increase of *RasGRP1* (*p* <0.001) and *RasGRP3* (*p* <0.01) gene expression levels after 48 hours of stimulation (Fig. 4a). To further investigate the potential role of TNFR1 and/or TNFR2 in TNFα-induced increase of *RasGRP1* or *RasGRP3* gene expression levels, we studied the effects of two antibodies neutralizing TNFR1 and TNFR2 specifically. First, we confirmed that TNFR1 and TNFR2 were expressed in B and T cells by flow cytometry (Fig. 4b). Second, the TNFR1 and TNFR2 neutralizing antibodies inhibited the TNFα-induced increase of *RasGRP1* (*p* <0.001) and *RasGRP3* (*p* <0.05) gene expression levels in T and B cells respectively (Fig. 4c). No effect was observed when T or B cells were treated only with TNFR1 or TNFR2 neutralizing antibodies (Additional file 6). No synergistic effect on *RasGRP1* and *RasGRP3* gene expression levels was observed when both TNFR1 and TNFR2 antibodies were simultaneously added to the cultures (data not shown). These experiments showed that TNFα modifies *RasGRP1* or *RasGRP3* expression *via* TNFR1 or TNFR2.

### TNFα influences RasGRP1 and RasGRP3 protein expression levels respectively in T and B cells

**Fig. 5:**
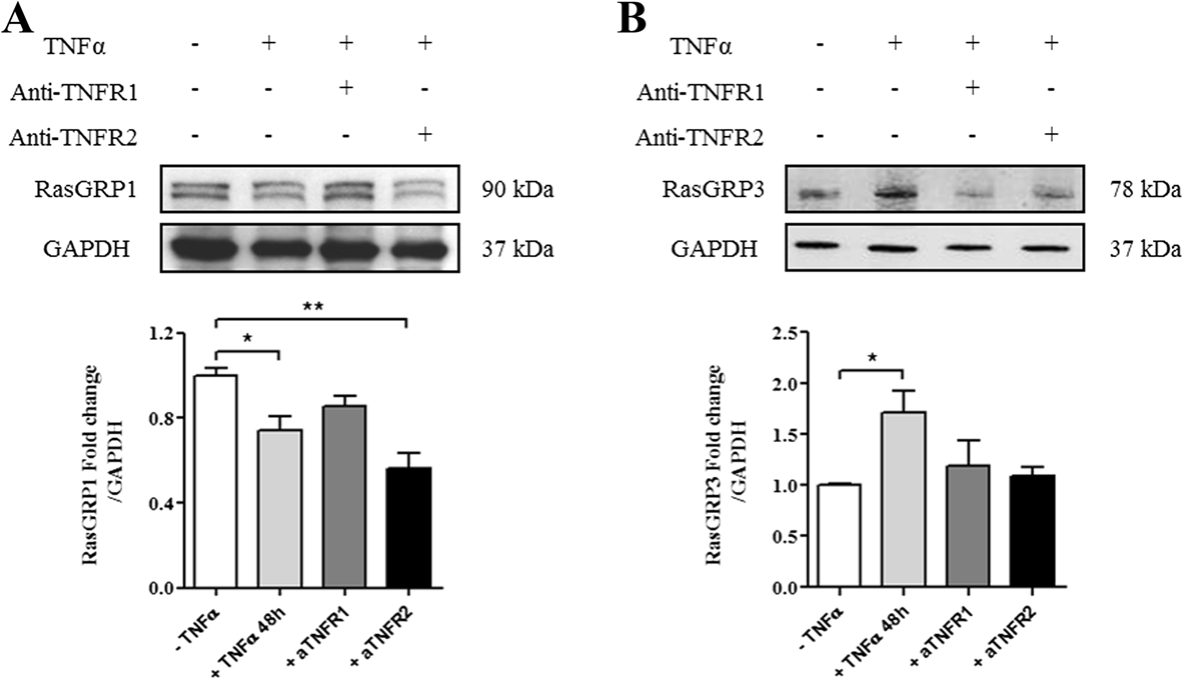
TNFα influences RasGRP1 and RasGRP3 protein expression levels respectively in T and B cells via TNF receptor (TNFR)1 and TNFR2. Western blot analysis was performed to measure RasGRP1 protein expression in T cells from five healthy controls (HC) (**a**) and RasGRP3 protein expression in B cells from three HC (**b**). In the control condition, cells were cultured without TNFα treatment. Cells were cultured with TNFα for 48 hours and with or without antibodies targeting TNFR1 or TNFR2. A value of 1 was arbitrarily assigned to control conditions (without TNFα treatment) to which glyceraldehyde-3-phosphate dehydrogenase (GAPDH) was reported and expressed as fold change. Mean ± standard error of the mean were compared using one-way analysis of variance followed by the Dunnett post-hoc test: *p <0.05; **p <0.01

In T cells, TNFα induced a significant decrease of RasGRP1 protein expression level after 48 hours of stimulation (*p* <0.05). As previously demonstrated in T cells from RA patients, the RasGRP1 gene (Fig. 4a) and protein (Fig. 5a) expressions changed in the opposite sense under TNFα stimulation. The use of TNFR1 neutralizing antibodies with TNFα had no effect on RasGRP1 expression while TNFR2 neutralizing antibodies with TNFα decrease RasGRP1 expression suggesting the participation of TNFR1 for RasGRP1 expression (Fig. 5a). In B cells, TNFα induced an increase of RasGRP3 protein expression level after 48 hours of stimulation (*p* <0.05) (Fig. 5b) but TNFα had no effect on RasGRP3 protein expression level after previous neutralization of TNFR1 or TNFR2 (Fig. 5b). Overall, TNFα increased the *RasGRP1* gene expression level while it decreased RasGRP1 protein expression independently of TNFR2. On the contrary, TNFα increased RasGRP3 mRNA and protein expression levels via TNFR1 or TNFR2.

### Functional effects of TNFα on T and B cells

**Fig. 6:**
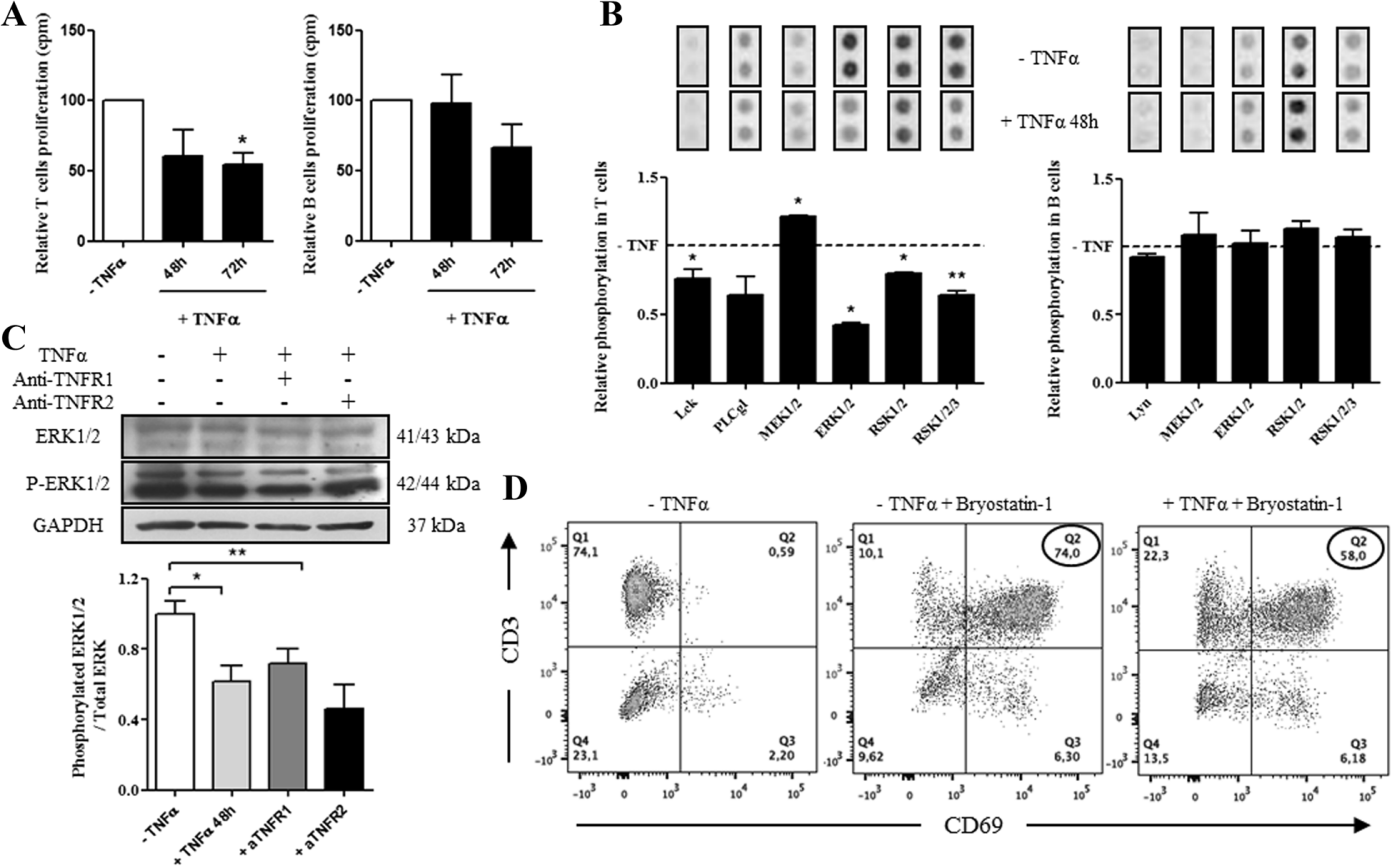
Functional effects of TNFα in B and T cells. **a** Cell proliferation was evaluated with (3H) thymidine incorporation by B and T cells with or without TNFα (n = 3). **b** Analysis of mitogen-activated protein kinase (MAPK) phosphorylation in B and T cells with or without TNFα for 48 hours was performed with a human phospho-kinase array kit to measure Lyn, Lck, PLCγ1, MEK, ERK and RSK phosphorylation (n = 2). The dotted line represents the control condition without TNFα. **c** Western blot analysis was performed to measure ERK1/2 and phospho-ERK1/2 protein expressions in T cells from five healthy controls (HC). Cells were cultured with or without TNFα for 48 hours and with or without antibodies targeting TNFR1 or TNFR2. *Histogram* represents ratio of phosphorylated ERK1/2 to total ERK. A value of 1 was arbitrarily assigned to control conditions (without TNFα treatment) to which glyceraldehyde-3-phosphate dehydrogenase (GAPDH) was reported and expressed as fold change. **d** After incubation with 1 ng/ml of TNFα for 48 hours, cells were stimulated with 50nM Bryostatin-1 for 5 hours. CD69 expression in T cells (CD3) was assessed using flow cytometry (one representative result of three independent experiments is presented). Mean ± standard error of the mean were compared using Student’s *t* test or one-way analysis of variance followed by Dunnett post-hoc test. *p <0.05; **p <0.01

RasGRP1 and RasGRP3 are involved respectively in TCR and BCR signaling pathways. TNFα modulates RasGRP1 and RasGRP3 in T and B cells, respectively. To date, no link between TNFR1 or TNFR2 and RasGRP1 or RasGRP3 has been described in the literature. Therefore, we characterized the TNFα effects in upstream and downstream of RasGRP1 and RasGRP3 on TCR and BCR signaling pathways. First, we evaluated cell proliferation under TNFα by (^3^H) thymidine incorporation. After 72 hours of stimulation, TNFα reduced proliferation only on T cells (*p* <0.05) (Fig. 6a) without apoptosis increasing (Additional file 7).

To better understand the decrease of T cell proliferation induced by TNFα and the putative link between RasGRP1 and TNFR1, a human phospho-kinase array kit was used to measure Lyn, Lck, phospholipase-Cγ1 (PLCγ1) (upstream of RasGRP), MEK, ERK and RSK (downstream of RasGRP) phosphorylation after 48 hours of TNFα stimulation. Indeed, the antigen binding to TCR or BCR, co-localized respectively with Lck and Lyn, induced RasGRP activation via the PLCγ1 [17, 18, 22]. The RasGRP proteins activate the small GTPase Ras resulting in the activation of the MAPK cascade Ras-Raf-MEK-ERK-RSK [18, 31]. TNFα induced significant inhibition of Lck, ERK and RSK phosphorylation (*p* <0.05) in T cells while TNFα had no significant effect on protein pathway phosphorylations in B cells (Fig. 6b). To confirm that TNFα inhibits ERK phosphorylation, T cells were incubated with or without TNFα and TNFR1 and TNFR2 neutralizing antibodies. TNFα induced a significant decrease of ERK phosphorylation after 48 hours of stimulation (*p* <0.05) (Fig. 6c). Whereas the use of TNFR1 neutralizing antibodies seems to inhibit the TNFα-induced decrease of ERK phosphorylation, the use of TNFR2 neutralizing antibodies had no effect on TNFα action (Fig. 6c). These results confirm the results obtained with RasGRP1 shown in Fig. 5a. So, TNFα induced a significant decrease of RasGRP1 protein expression level after 48 hours of stimulation, followed by an inhibition of ERK phosphorylation and T cell proliferation (Fig. 6). To confirm that inhibition of ERK phosphorylation and T cell proliferation is due to the decrease of RasGRP1 expression, T cells were stimulated with Bryostatin-1 (an activator of RasGRP1) for 5 hours after TNFα incubation. CD69 expression (a marker of T cell activation) was measured by flow cytometry. Bryostatin-1 induced CD69 expression in human T cells (Fig. 6d, left and middle panels). However, TNFα inhibited the increase of CD69 expression induced by Bryostatin-1 (74 % versus 58 %) (Fig. 6d, right panel). In conclusion, TNFα binding to TNFR1 inhibits RasGRP1 expression, followed by inhibition of ERK phosphorylation and subsequent decrease in T cell proliferation.

## Discussion

Previous studies conducted in our laboratory have identified *RasGRP3* as a biomarker of TNFα inhibitor (infliximab) response and RA synovial tissue [8, 9]. These observations and the literature analysis lead us to wonder whether RasGRP family proteins have a role in RA. As RasGRP2 and RasGRP4 are expressed respectively in neuronal cells and mast cells [13, 21], we focused our study on RasGRP1 and RasGRP3 expression involved respectively in TCR and BCR signaling.

We demonstrated the preferential expression of *RasGRP1* and *RasGRP3* mRNAs, respectively, in human T and B cells. The other data available on RasGRP1 and RasGRP3 are derived from experimental studies in mice, from cell lines and from systemic lupus erythematosus (SLE) T cells [12, 20]. These experiments showed that in mice RasGRP1 is expressed in B and T cells whereas RasGRP3 is expressed only in B cells. Therefore, our results in humans are in accordance with the experimental data on the expression levels of these proteins in lymphocytes from mice. In addition, in Jurkat T-cell-lines, RasGRP1 maintains expression of TCRα mRNA and surface expression of the TCR/CD3 complex suggesting a regulatory function of RasGRP1 signal in peripheral T cells [32]. A subset of SLE patients have defective expression of *RasGRP1* because of aberrant splicing. To rule out the hypothesis that the modulation of *RasGRP1* transcript level is due to different isoforms of *RasGRP1* identified by Yasuda et al. [29], we measured the expression level of the different isoforms present in RNA samples from 18 RA patients. We found no substantial difference in the levels of the normal, full-length *RasGRP1* transcript in these cells relative to that of the cells from HC (data not shown). Moreover, the primers chosen for *RasGRP1* qRT-PCR were located upstream of exon 11, the exon preferentially deleted in SLE patients and determined by Yasuda et al. [29].

In our study, the analysis of RasGRP1 and RasGRP3 expression levels showed only upregulation of RasGRP1 in T cells from RA patients compared to HC (Fig. 2b). Recent genome-wide association studies have identified *RasGRP1* as a gene of susceptibility in RA [10], which supports the dysregulation of RasGRP1 in RA. Moreover, *RasGRP1* gene expression was also correlated with RA disease activity (Fig. 2d) as was observed in SLE [33]. But *RasGRP1* and disease activity were inversely correlated in RA while they were positively correlated in SLE suggesting different ways of regulation in these two diseases. Furthermore, in T cells from RA patients compared with those of HC, *RasGRP1* gene expression level was increased in T cells (Fig. 2b) while RasGRP1 protein expression was reduced (Fig. 3). However, T cell activation experiments showed that the activation status of these cells sharply increases the *RasGRP1* gene expression level (Additional file 5B). So, the increased *RasGRP1* gene expression level observed in RA patients could reflect the T cell activation status occurring in this inflammatory context. Moreover, these opposing observations could be explained by the presence of microRNA-21 targeting *RasGRP1* mRNA. However, microRNA-21 was previously highlighted as being overexpressed in SLE and RA patients [34, 35].

In vitro studies with T cells from HC showed us that TNFα stimulation (a key cytokine in RA pathophysiology) increased *RasGRP1* gene expression level (Fig. 4a) while it reduced RasGRP1 protein expression level (Fig. 5a). This discrepancy could be explained by the presence of microRNA-21. Indeed, several studies have demonstrated that microRNA-21 could be upregulated by TNFα in cancer or in some inflammatory pathology [36–39]. These data are in agreement with the results found in the *RasGRP1*^*-/-*^ mouse model, in which mice became autoimmune-prone and developed a lupus-like phenotype [20]. Indeed, this study demonstrated that RasGRP1 inhibition instigates the emergence of autoimmunity. However, TNFα did not induce modulation of *RasGRP1* gene expression level in PBMCs from RA patients (Fig. 1a). The hypothesis would be that the addition of TNFα in vitro to PBMCs from RA patients, already in an inflammatory environment, has no effect on *RasGRP1* gene expression level. This phenomenon is even more reinforced by the absence of an effect of TNFα inhibitors on *RasGRP1* gene expression level (Fig. 1a). Whereas the use of TNFR1 neutralizing antibody inhibited the TNFα-induced decreasing of RasGRP1 expression level, the use of TNFR2 neutralizing antibody had no effect on TNFα action (Fig. 5a). These results could be explained by the fact that the TNFR1 binds soluble TNFα, whereas TNFR2 binds only the membrane TNFα. In accordance with these results, we demonstrated inhibition of T cell proliferation (Fig. 6a) and also significant inhibition of Lck, ERK and RSK phosphorylation after TNFα treatment (Fig. 6b). We also demonstrated that TNFα inhibited ERK phosphorylation and only the use of TNFR1 neutralizing antibody inhibited this TNFα effect (Fig. 6c). Moreover, bryostatin-1 experiments have confirmed that the TNFα effect observed in T cell proliferation and ERK phosphorylation was indeed due to the decrease of RasGRP1 expression (Fig. 6d).

To better understand RasGRP3 function, we measured *RasGRP1* and *RasGRP3* gene expression levels in patients treated by adalimumab and etanercept (TNFα inhibitors), and abatacept, a CTLA4-Ig fusion protein as control. *RasGRP3* mRNA abundance was decreased only in PBMCs from RA patients treated by TNFα inhibitors but was increased in PBMCs from RA patients treated with abatacept. Furthermore, *RasGRP3* gene expression levels were not correlated with DAS28 score (data not shown) and RasGRP3 was not dysregulated in RA patients compared to HC (Figs. 2c and 3b). On the contrary, *RasGRP3* gene expression level increased two-fold in PBMCs from RA patients (Fig. 1b) and six-fold in B cells from HC after 48 hours of culture with TNFα (Figs. 4a and 5b), leading us to conclude that RasGRP3 in B cells was modulated by TNFα. So, it is not surprising to find *RasGRP3* as a biomarker of responsiveness to TNFα inhibitors [8]. TNFα induced RasGRP3 increase via TNFR1 and TNFR2. These results are in accordance with the effects of TNFα inhibitors on *RasGRP3* gene expression level. Indeed, patients treated by TNFα inhibitors display a decrease of *RasGRP3* gene expression level. So, the inhibition of *RasGRP3* gene expression seems to be of good prognosis for RA patients. In addition, double mutant mice *RasGRP1*^*-/-*^*RasGRP3*^*-/-*^ do not develop signs of autoimmunity contrary to *RasGRP1*^*-/-*^ mice [12]. Therefore, RasGRP3 is a biomarker related to TNFα inhibitors.

Aberrant splicing and expression of RasGRP4 were discovered respectively in PBMCs and in the fibroblast-like synoviocytes (FLS) of a subset of RA patients [40, 41]. The defective transcripts led to alteration of the MAPK pathway in PBMCs while the level of *RasGRP4* transcript was correlated with the FLS proliferation rate [40, 41]. Any aberrant splicing of *RasGRP1* or *RasGRP3* was found in our RA patients (data not shown). In experimental arthritis, RasGRP4-null mice did not develop K/BxN inflammatory arthritis and intra-articular injection of RasGRP4-specific siRNAs reduced the severity of the disease, suggesting that RasGRP4 is a potential target of therapy in RA [41]. Moreover, RasGRPs represent potential therapeutic targets in cancer [42]. Taken together, the study on RasGRP4 and our study focused on RasGRP1 and RasGRP3 bring new insights into a regulatory function of the RasGRP protein family in FLS and/or in immune cells from RA patients, thereby representing a potential target in RA.

## Conclusion

In summary, our study demonstrated the dysregulation of RasGRP1 in RA patients and the role of RasGRP3 as a biomarker of adalimumab and etanercept. This study suggests a link in T and B cells, never described previously, between RasGRP1 or RasGRP3 and TNFα. While the response to TNFα inhibitors in RA patients modulates *RasGRP3* gene expression, TNFα inhibits RasGRP1 protein expression, leading to TCR pathway inhibition. We can speculate the establishment of a negative feedback by TNFα to inhibit T cell activation, via RasGRP1 inhibition (Additional file 8). Taken together, the recent study on RasGRP4 and these new data focused on RasGRP1 and RasGRP3 bring new insights into a regulatory function of the RasGRP protein family in FLS and/or in immune cells from RA patients, thereby representing a potential target in RA.

## Additional files

Additional file 1: Table S1. Clinical and biological characteristics of rheumatoid arthritis (*RA*) patients treated with adalimumab, etanercept or abatacept. (DOC 61 kb) Additional file 2: Table S2. Clinical features of patients and healthy controls. (DOC 40 kb) Additional file 3:
**Modulation of** ***RasGRP3*** **gene expression levels in peripheral blood mononuclear cells (PBMCs) from healthy controls (HC) incubated with TNFα and adalimumab, etanercept, infliximab, certolizumab or golimumab.** Quantitative PCR analysis was performed to measure *RasGRP3* gene expression levels in PBMCs obtained from HC treated by TNFα and TNFα inhibitors. In each condition, cells were cultured with or without TNFα for 48 hours and adalimumab (n = 4) (**a**), etanercept (n = 3) (**b**), infliximab (n = 3) (**c**), certolizumab (n = 3) (**d**) or golimumab (n = 3) (**e**) was added. The relative expression levels (in arbitrary units (*AU*)) of *RasGRP3* were normalized with *18S* RNA abundance. Mean ± standard error of the mean were compared using one-way analysis of variance followed by Bonferroni post-hoc test: **p* <0.05; ***p* <0.01; ****p* <0.001. (TIF 1307 kb) Additional file 4:
**T and B cell purity.** After negative selection of peripheral blood mononuclear cells from healthy controls, buffy coat and rheumatoid arthritis patients, cytometric analysis of T and B cell purity was checked. **a** Cytometric analysis of human T cell purity with CD3, CD4 and CD8 labeling. **b** Cytometric analysis of human B cell purity with CD19 labeling. (TIF 1096 kb) Additional file 5:
**T cell activation induces an increase of** ***RasGRP1*** **gene expression level.** After T cell negative selection, cells were cultured with IL-2 and anti-CD3 antibody for 4 days (n = 3). **a** To evaluate the T cell activation status, HLA-DR and CD25 expressions in T cells (CD3) was checked by flow cytometry. **b** quantitative PCR analysis of *RasGRP1* gene expression was performed. The relative expression levels (in arbitrary units (AU)) of *RasGRP1* were normalized with *18S* RNA abundance. Mean ± standard error of the mean were compared using Student’s *t* test: ****p* <0.001. (TIF 100 kb) Additional file 6:
**TNF receptor (TNFR)1 and TNFR2 have no effect on** ***RasGRP1*** **and** ***RasGRP3*** **gene expression level in T and B cells respectively.** Quantitative PCR analysis was performed to measure *RasGRP1* and *RasGRP3* gene expression levels in T and B cells respectively from three healthy controls. T or B cells were exposed to anti-TNFR1 or anti-TNFR2 neutralizing antibodies without TNFα for 48 hours. The relative expression levels (in arbitrary units (*AU*)) of *RasGRP1* and *RasGRP3* were normalized with *18S* RNA abundance. Mean ± standard error of the mean were compared using one-way analysis of variance followed by Bonferroni post-hoc test. (TIF 47 kb) Additional file 7:
**TNFα has no effect on T cell apoptosis.** After T cell negative selection, cells were cultured with or without TNFα for 48 and 72 hours. To measure apoptosis, a fluorescein isothiocyanate (FITC) annexin-V Apoptosis Detection Kit (BD Biosciences, USA) was used. After labeling with FITC annexin-V and propidium iodide (*PI*), cells were analyzed by flow cytometry within 1 hour. **a** One representative result of three independent experiments is presented. **b** *Histogram* represents the mean ± standard error of the mean (SEM) of percentage of annexin-V positive cells in three independent experiments. Mean ± SEM were compared using Student’s *t* test. (TIF 1318 kb) Additional file 8:
**A model of RasGRP1 regulation by TNFα. In T cells, TNFα binding to TNF receptor-1** (***TNFR1***) **leads to a decrease of RasGRP1 protein expression after 48 hours of stimulation.** This mechanism prevents mitogen-activated protein kinase activation and induces inhibition of T cell activation and proliferation. We can speculate the establishment of a negative feedback by TNFα to inhibit T cell activation after 48 hours of stimulation, via RasGRP1 inhibition. (TIF 869 kb)

## Abbreviations

Ab: Antibody
ACR: American College of Rheumatology
ANSM: French National Agency for Medicines
ANOVA: analysis of variance
BCR: B cell receptor
BSA: bovine serum albumin
DAG: DiAcylGlycerol
DAS28: disease activity score 28
ERK: extracellular signal-regulated kinase
FCS: fetal calf serum
FLS: fibroblast-like synoviocyte
GAPDH: glyceraldehyde-3-phosphate dehydrogenase
HC: healthy controls
IL: interleukin
MAPK: mitogen-activated protein kinase
mRNA: messenger ribonucleic acid
NF-κB: nuclear factor-kappa B
PBMC: peripheral blood mononuclear cell
PBS: phosphate-buffered saline
PLC γ1: phospholipase-Cγ1
qRT-PCR: quantitative real-time polymerase chain reaction
RA: rheumatoid arthritis
RasGRP: ras guanyl nucleotide-releasing protein
RNA: ribonucleic acid
RPMI: Roswell Park Memorial Institute
SDS: sodium dodecyl sulfate
SLE: systemic lupus erythematosus
TCR: T cell receptor
TNFR: tumor necrosis factor receptor
TNFα: tumor necrosis factor α

## References

[1] Hazes JM, Silman AJ (1990). Review of UK data on the rheumatic diseases–2. Rheumatoid arthritis. Br J Rheumatol..

[2] Arend WP, Firestein GS (2012). Pre-rheumatoid arthritis: predisposition and transition to clinical synovitis. Nat Rev Rheumatol..

[3] Boissier MC, Semerano L, Challal S, Saidenberg-Kermanac'h N, Falgarone G (2012). Rheumatoid arthritis: from autoimmunity to synovitis and joint destruction. J Autoimmun..

[4] McInnes IB, Schett G (2007). Cytokines in the pathogenesis of rheumatoid arthritis. Nat Rev Immunol..

[5] Brennan FM, McInnes IB (2008). Evidence that cytokines play a role in rheumatoid arthritis. J Clin Invest..

[6] Keffer J, Probert L, Cazlaris H, Georgopoulos S, Kaslaris E, Kioussis D (1991). Transgenic mice expressing human tumour necrosis factor: a predictive genetic model of arthritis. EMBO J..

[7] Singh JA, Christensen R, Wells GA, Suarez-Almazor ME, Buchbinder R, Lopez-Olivo MA (2010). Biologics for rheumatoid arthritis: an overview of Cochrane reviews. Sao Paulo Med J..

[8] Lequerré T, Gauthier-Jauneau AC, Bansard C, Derambure C, Hiron M, Vittecoq O (2006). Gene profiling in white blood cells predicts infliximab responsiveness in rheumatoid arthritis. Arthritis Res Ther..

[9] Lequerré T, Bansard C, Vittecoq O, Derambure C, Hiron M, Daveau M (2009). Early and long-standing rheumatoid arthritis: distinct molecular signatures identified by gene-expression profiling in synovia. Arthritis Res Ther..

[10] Eyre S, Bowes J, Diogo D, Lee A, Barton A, Martin P (2012). High-density genetic mapping identifies new susceptibility loci for rheumatoid arthritis. Nat Genet..

[11] Ebinu JO, Bottorff DA, Chan EY, Stang SL, Dunn RJ, Stone JC (1998). RasGRP, a Ras guanyl nucleotide-releasing protein with calcium- and diacylglycerol-binding motifs. Science..

[12] Coughlin JJ, Stang SL, Dower NA, Stone JC (2005). RasGRP1 and RasGRP3 regulate B cell proliferation by facilitating B cell receptor-Ras signaling. J Immunol..

[13] Stone JC (2006). Regulation of Ras in lymphocytes: get a GRP. Biochem Soc Trans..

[14] Zheng Y, Liu H, Coughlin J, Zheng J, Li L, Stone JC (2005). Phosphorylation of RasGRP3 on threonine 133 provides a mechanistic link between PKC and Ras signaling systems in B cells. Blood..

[15] Aiba Y, Oh-hora M, Kiyonaka S, Kimura Y, Hijikata A, Mori Y (2004). Activation of RasGRP3 by phosphorylation of Thr-133 is required for B cell receptor-mediated Ras activation. Proc Natl Acad Sci USA.

[16] Teixeira C, Stang SL, Zheng Y, Beswick NS, Stone JC (2003). Integration of DAG signaling systems mediated by PKC-dependent phosphorylation of RasGRP3. Blood..

[17] Liu SK, Berry DM, McGlade CJ (2001). The role of Gads in hematopoietic cell signalling. Oncogene..

[18] Dal Porto JM, Gauld SB, Merrell KT, Mills D, Pugh-Bernard AE, Cambier J (2004). B cell antigen receptor signaling 101. Mol Immunol..

[19] Guilbault B, Kay RJ (2004). RasGRP1 sensitizes an immature B cell line to antigen receptor-induced apoptosis. J Biol Chem..

[20] Coughlin JJ, Stang SL, Dower NA, Stone JC (2006). The role of RasGRPs in regulation of lymphocyte proliferation. Immunol Lett..

[21] Stone JC (2011). Regulation and Function of the RasGRP Family of Ras Activators in Blood Cells. Genes Cancer..

[22] Bivona TG, Perez De Castro I, Ahearn IM, Grana TM, Chiu VK, Lockyer PJ (2003). Phospholipase Cgamma activates Ras on the Golgi apparatus by means of RasGRP1. Nature.

[23] Dower NA, Stang SL, Bottorff DA, Ebinu JO, Dickie P, Ostergaard HL (2000). RasGRP is essential for mouse thymocyte differentiation and TCR signaling. Nat Immunol..

[24] Arnett FC, Edworthy SM, Bloch DA, McShane DJ, Fries JF, Cooper NS (1987). revised criteria for the classification of rheumatoid arthritis. Arthritis Rheum.

[25] Aletaha D, Neogi T, Silman AJ, Funovits J, Felson DT, Bingham CO (2010). 2010 Rheumatoid arthritis classification criteria: an American College of Rheumatology/European League Against Rheumatism collaborative initiative. Arthritis Rheum..

[26] D’Agostino MA, Wakefield R, Berner Hammer H, Vittecoq O, Galeazzi M, Balint P (2012). Assessment of omeract global power doppler ultrasonography 44-joint scoring system and reduced joint scoring systems in rheumatoid arthritis patients treated with abatacept plus background methotrexate [abstract]. Arthritis Rheum.

[27] Poltorak M, Meinert I, Stone JC, Schraven B, Simeoni L (2014). Sos1 regulates sustained TCR-mediated Erk activation. Eur J Immunol..

[28] Roose JP, Mollenauer M, Ho M, Kurosaki T, Weiss A (2007). Unusual interplay of two types of Ras activators, RasGRP and SOS, establishes sensitive and robust Ras activation in lymphocytes. Mol Cell Biol..

[29] Yasuda S, Stevens RL, Terada T, Takeda M, Hashimoto T, Fukae J (2007). Defective expression of Ras guanyl nucleotide-releasing protein 1 in a subset of patients with systemic lupus erythematosus. J Immunol..

[30] Ksionda O, Limnander A, Roose JP (2013). RasGRP Ras guanine nucleotide exchange factors in cancer. Front Biol..

[31] Ebinu JO, Stang SL, Teixeira C, Bottorff DA, Hooton J, Blumberg PM (2000). RasGRP links T-cell receptor signaling to Ras. Blood..

[32] Markegard E, Trager E, Yang CW, Zhang W, Weiss A, Roose JP (2011). Basal LAT-diacylglycerol-RasGRP1 signals in T cells maintain TCRα gene expression. PLoS One..

[33] Rapoport MJ, Bloch O, Amit-Vasina M, Yona E, Molad Y (2011). Constitutive abnormal expression of RasGRP-1 isoforms and low expression of PARP-1 in patients with systemic lupus erythematosus. Lupus..

[34] Pan W, Zhu S, Yuan M, Cui H, Wang L, Luo X (2010). MicroRNA-21 and microRNA-148a contribute to DNA hypomethylation in lupus CD4+ T cells by directly and indirectly targeting DNA methyltransferase 1. J Immunol..

[35] Wang H, Peng W, Ouyang X, Li W, Dai Y (2012). Circulating microRNAs as candidate biomarkers in patients with systemic lupus erythematosus. Transl Res..

[36] Cottonham CL, Kaneko S, Xu L (2010). miR-21 and miR-31 converge on TIAM1 to regulate migration and invasion of colon carcinoma cells. J Biol Chem.

[37] Guinea-Viniegra J, Jiménez M, Schonthaler HB, Navarro R, Delgado Y, Concha-Garzón MJ (2014). Targeting miR-21 to treat psoriasis. Sci Transl Med..

[38] Kagiya T, Nakamura S (2013). Expression profiling of microRNAs in RAW264.7 cells treated with a combination of tumor necrosis factor alpha and RANKL during osteoclast differentiation. J Periodontal Res.

[39] Zarjou A, Yang S, Abraham E, Agarwal A, Liu G (2011). Identification of a microRNA signature in renal fibrosis: role of miR-21. Am J Physiol Renal Physiol..

[40] Hashimoto T, Yasuda S, Koide H, Kataoka H, Horita T, Atsumi T (2011). Aberrant splicing of the hRasGRP4 transcript and decreased levels of this signaling protein in the peripheral blood mononuclear cells in a subset of patients with rheumatoid arthritis. Arthritis Res Ther..

[41] Kono M, Yasuda S, Stevens RL, Koide H, Kurita T, Shimizu Y (2015). Ras guanine nucleotide-releasing protein 4 is aberrantly expressed in the fibroblast-like synoviocytes of patients with rheumatoid arthritis and controls their proliferation. Arthritis Rheumatol..

[42] Song X, Lopez-Campistrous A, Sun L, Dower NA, Kedei N, Yang J (2013). RasGRPs are targets of the anti-cancer agent ingenol-3-angelate. PLoS One..

