# Supplementary material for: Dysregulation of RasGRP1 in rheumatoid arthritis and modulation of RasGRP3 as a biomarker of TNFα inhibitors
Source: Arthritis Res Ther. 2015 Dec 26;17:382. doi: 10.1186/s13075-015-0894-9 (PMC4718016; doi:10.1186/s13075-015-0894-9)
Supplement: Additional file 1: Table S1. — Clinical and biological characteristics of rheumatoid arthritis (RA) patients treated with adalimumab, etanercept or abatacept. (DOC 61 kb) [file 13075_2015_894_MOESM1_ESM.doc]

**Additional file 1: Table S1: Clinical and biological characteristics of RA patients treated with adalimumab, etanercept or abatacept.**

**
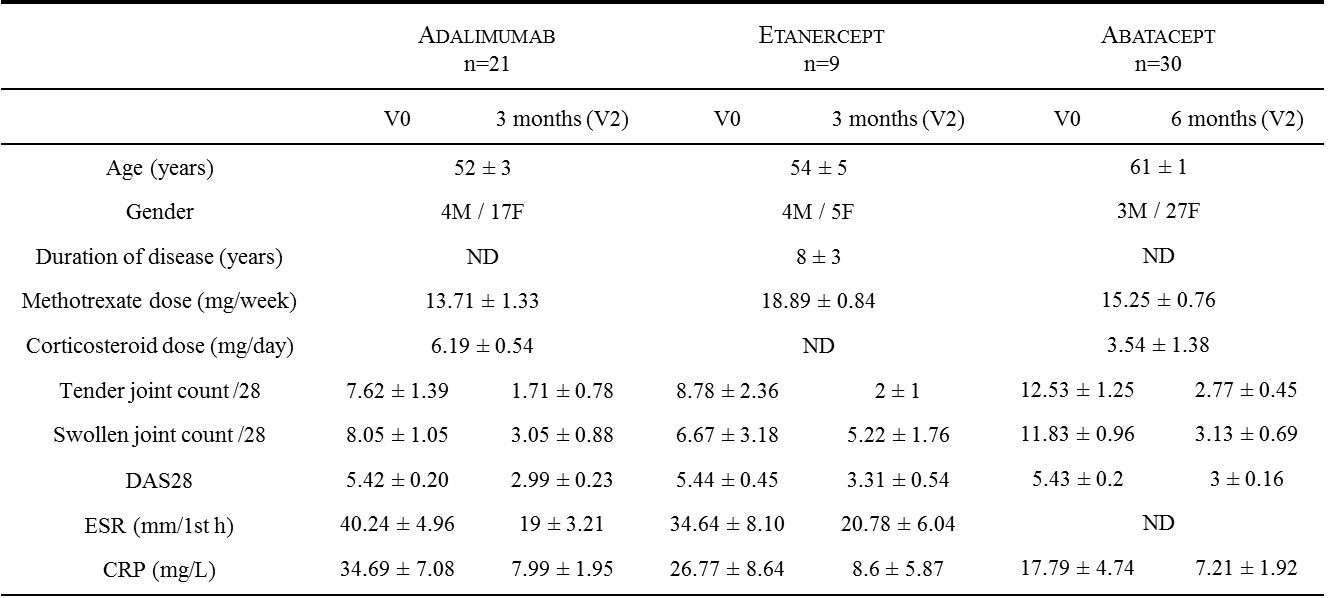
**

Values are presented as mean ± standard error of the mean (SEM). CRP: C reactive protein; DAS28: disease activity score; ESR: erythrocyte sedimentation rate; ND: not determined.
