# Supplementary material for: Dysregulation of RasGRP1 in rheumatoid arthritis and modulation of RasGRP3 as a biomarker of TNFα inhibitors
Source: Arthritis Res Ther. 2015 Dec 26;17:382. doi: 10.1186/s13075-015-0894-9 (PMC4718016; doi:10.1186/s13075-015-0894-9)
Supplement: Additional file 2: Table S2. — Clinical features of patients and healthy controls. (DOC 40 kb) [file 13075_2015_894_MOESM2_ESM.doc]

**Additional file 2: Table S2: Clinical features of patients and HC.**

**
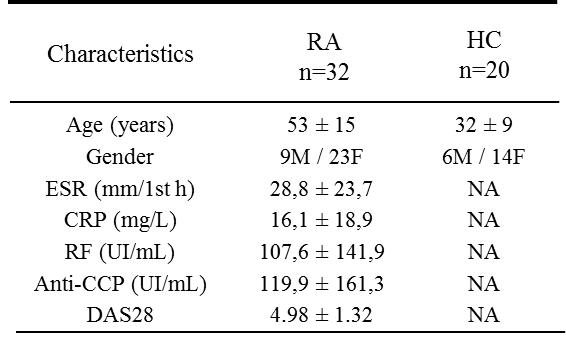
**

Values are presented as mean ± standard error of the mean (SEM). anti-CCP: anti-cyclic citrullinated peptide; CRP: C reactive protein; DAS28: disease activity score; ESR: erythrocyte sedimentation rate; HC: healthy controls; NA: not applicable; RA: rheumatoid arthritis; RF: rheumatoid factor.
