# Supplementary figures and images for: Dysregulation of RasGRP1 in rheumatoid arthritis and modulation of RasGRP3 as a biomarker of TNFα inhibitors
Source: Arthritis Res Ther. 2015 Dec 26;17:382. doi: 10.1186/s13075-015-0894-9 (PMC4718016; doi:10.1186/s13075-015-0894-9)

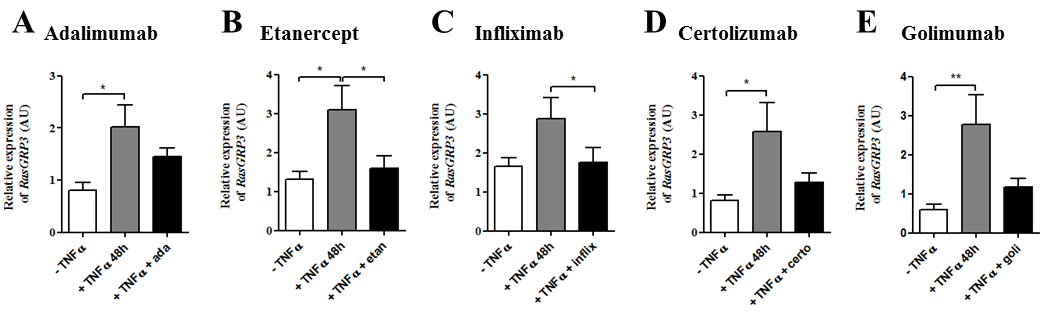

Supplement: Additional file 3: — Modulation of RasGRP3 gene expression levels in peripheral blood mononuclear cells (PBMCs) from healthy controls (HC) incubated with TNFα and adalimumab, etanercept, infliximab, certolizumab or golimumab. Quantitative PCR analysis was performed to measure RasGRP3 gene expression levels in PBMCs obtained from HC treated by TNFα and TNFα inhibitors. In each condition, cells were cultured with or without TNFα for 48 hours and adalimumab (n = 4) (a), etanercept (n = 3) (b), infliximab (n = 3) (c), certolizumab (n = 3) (d) or golimumab (n = 3) (e) was added. The relative expression levels (in arbitrary units (AU)) of RasGRP3 were normalized with 18S RNA abundance. Mean ± standard error of the mean were compared using one-way analysis of variance followed by Bonferroni post-hoc test: *p <0.05; **p <0.01; ***p <0.001. (TIF 1307 kb) [file 13075_2015_894_MOESM3_ESM.tif]

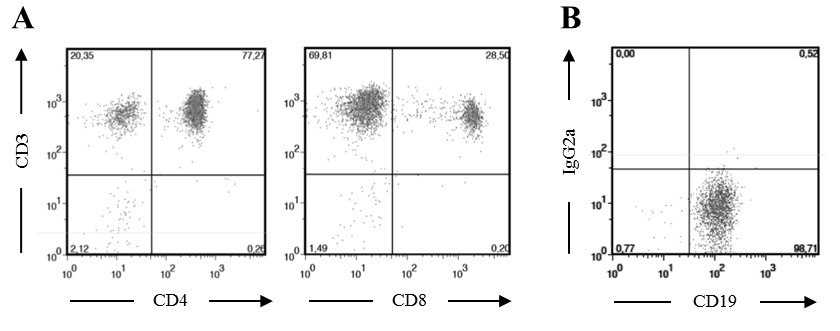

Supplement: Additional file 4: — T and B cell purity. After negative selection of peripheral blood mononuclear cells from healthy controls, buffy coat and rheumatoid arthritis patients, cytometric analysis of T and B cell purity was checked. a Cytometric analysis of human T cell purity with CD3, CD4 and CD8 labeling. b Cytometric analysis of human B cell purity with CD19 labeling. (TIF 1096 kb) [file 13075_2015_894_MOESM4_ESM.tif]

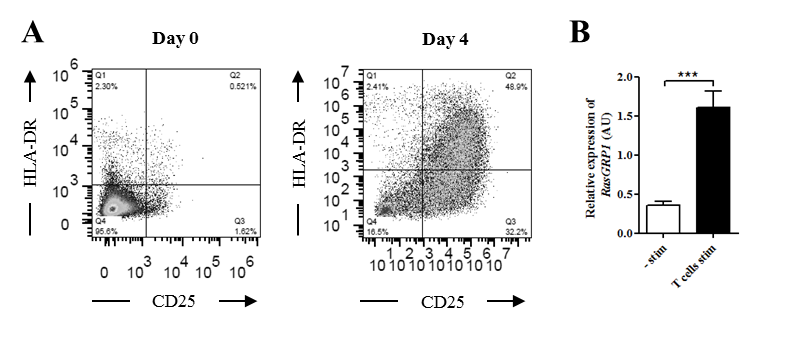

Supplement: Additional file 5: — T cell activation induces an increase of RasGRP1 gene expression level. After T cell negative selection, cells were cultured with IL-2 and anti-CD3 antibody for 4 days (n = 3). a To evaluate the T cell activation status, HLA-DR and CD25 expressions in T cells (CD3) was checked by flow cytometry. b quantitative PCR analysis of RasGRP1 gene expression was performed. The relative expression levels (in arbitrary units (AU)) of RasGRP1 were normalized with 18S RNA abundance. Mean ± standard error of the mean were compared using Student’s t test: ***p <0.001. (TIF 100 kb) [file 13075_2015_894_MOESM5_ESM.tif]

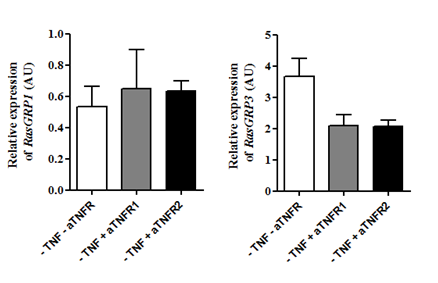

Supplement: Additional file 6: — TNF receptor (TNFR)1 and TNFR2 have no effect on RasGRP1 and RasGRP3 gene expression level in T and B cells respectively. Quantitative PCR analysis was performed to measure RasGRP1 and RasGRP3 gene expression levels in T and B cells respectively from three healthy controls. T or B cells were exposed to anti-TNFR1 or anti-TNFR2 neutralizing antibodies without TNFα for 48 hours. The relative expression levels (in arbitrary units (AU)) of RasGRP1 and RasGRP3 were normalized with 18S RNA abundance. Mean ± standard error of the mean were compared using one-way analysis of variance followed by Bonferroni post-hoc test. (TIF 47 kb) [file 13075_2015_894_MOESM6_ESM.tif]

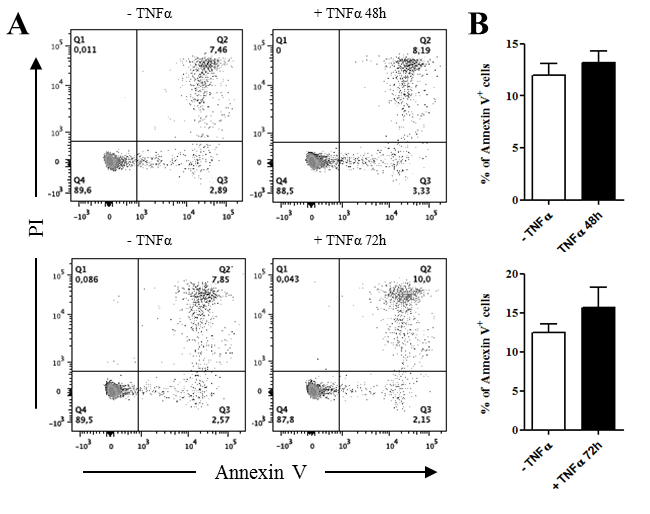

Supplement: Additional file 7: — TNFα has no effect on T cell apoptosis. After T cell negative selection, cells were cultured with or without TNFα for 48 and 72 hours. To measure apoptosis, a fluorescein isothiocyanate (FITC) annexin-V Apoptosis Detection Kit (BD Biosciences, USA) was used. After labeling with FITC annexin-V and propidium iodide (PI), cells were analyzed by flow cytometry within 1 hour. a One representative result of three independent experiments is presented. b Histogram represents the mean ± standard error of the mean (SEM) of percentage of annexin-V positive cells in three independent experiments. Mean ± SEM were compared using Student’s t test. (TIF 1318 kb) [file 13075_2015_894_MOESM7_ESM.tif]

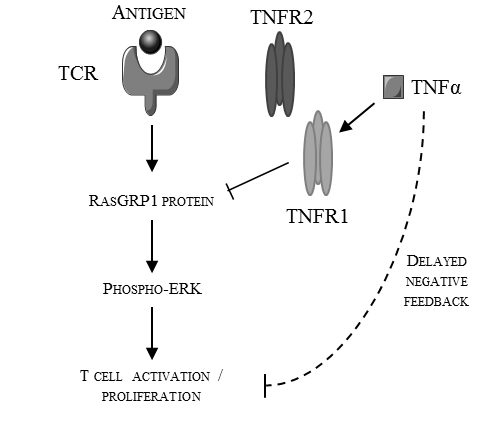

Supplement: Additional file 8: — A model of RasGRP1 regulation by TNFα. In T cells, TNFα binding to TNF receptor-1 (TNFR1) leads to a decrease of RasGRP1 protein expression after 48 hours of stimulation. This mechanism prevents mitogen-activated protein kinase activation and induces inhibition of T cell activation and proliferation. We can speculate the establishment of a negative feedback by TNFα to inhibit T cell activation after 48 hours of stimulation, via RasGRP1 inhibition. (TIF 869 kb) [file 13075_2015_894_MOESM8_ESM.tif]
